# Supplementary material for: Genomic innovations linked to infection strategies across emerging pathogenic chytrid fungi
Source: Nat Commun. 2017 Mar 21;8:14742. doi: 10.1038/ncomms14742 (PMC5364385; doi:10.1038/ncomms14742)
Supplement: Supplementary Information — Supplementary Figures and Supplementary Tables [file ncomms14742-s1.pdf]

Supplementary Figures

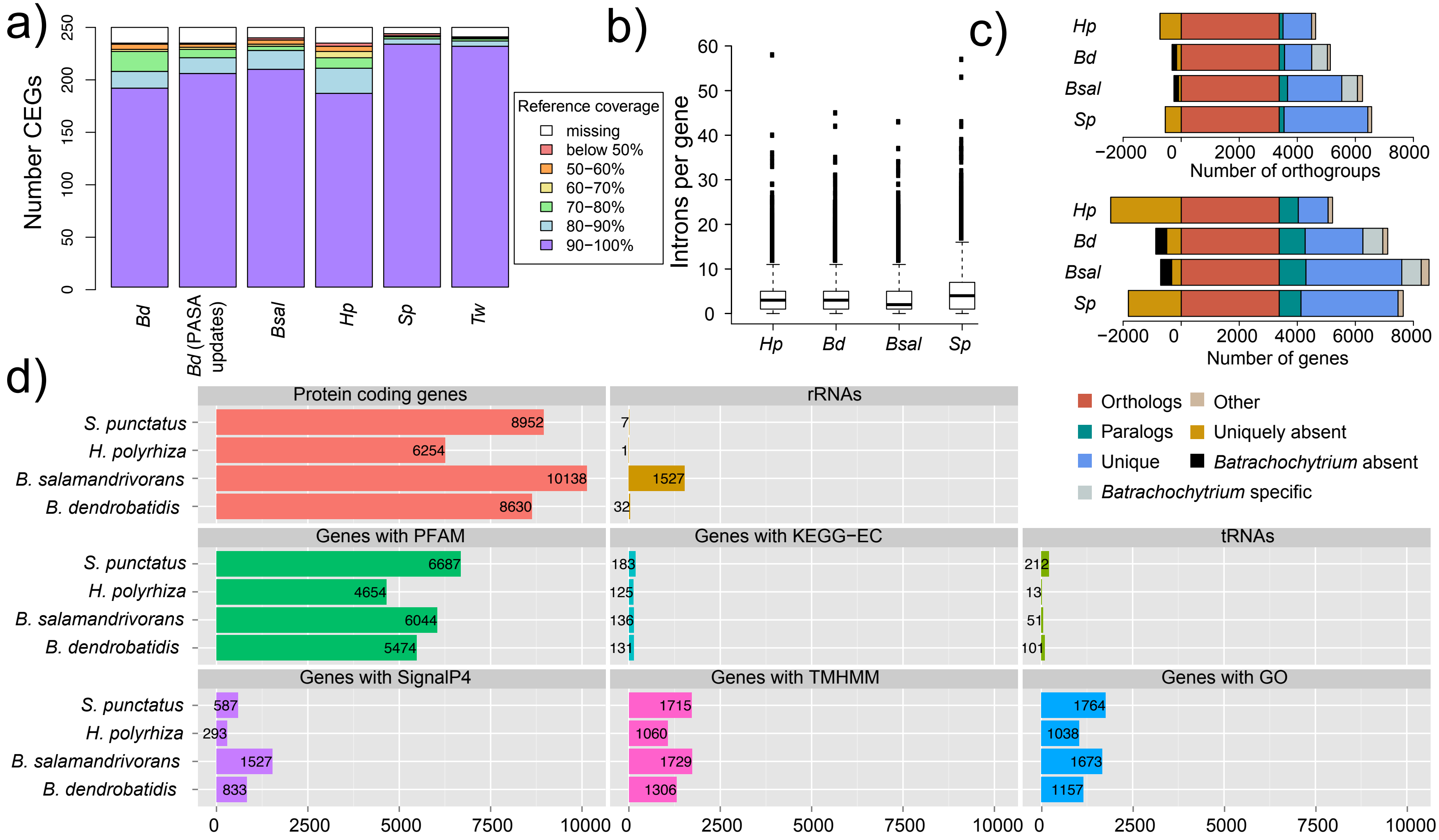

Supplementary Figure 1 Assessments of the Chytridiomycota gene calls. (**a**) All gene sets were compared against 248 core eukaryotic genes (CEGs), indicating a highly complete set to work with, as well as a substantial improvement for *Bd* using RNA-Seq data. (**b**) The number of introns per gene set. (**c**) The number of orthogroups (shown above), and the number of genes in each category (shown below). (**d**) Number of protein coding genes, rRNAs, tRNAs, and the number of protein coding genes with functional annotation (PFAM, KEGG-EC, GO, SignalP 4.0 and TMHMM

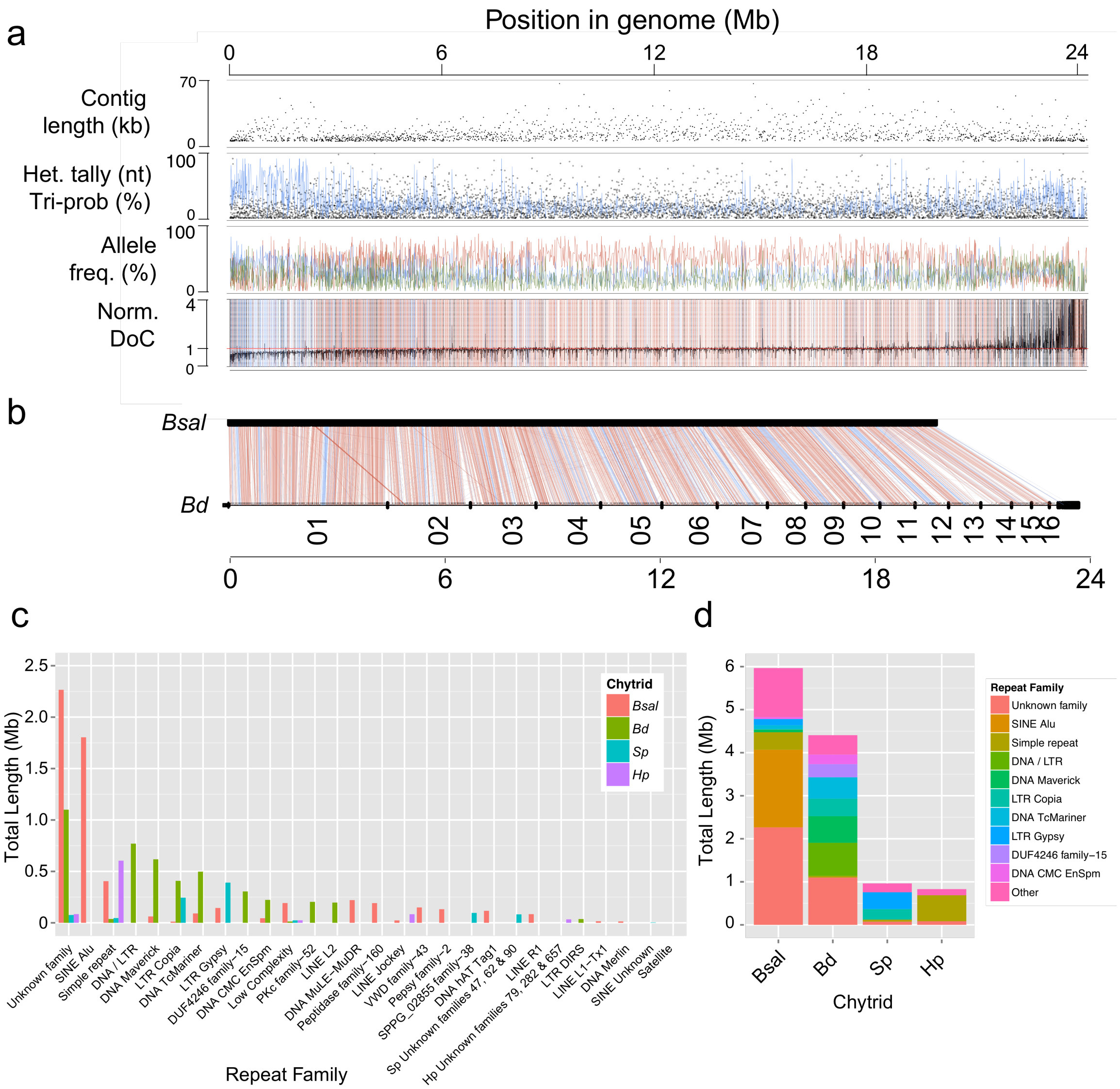

Supplementary Figure 2 Assessments of the Chytridiomycota ploidy and repetitive content. (a) Bi-allelic heterozygous positions in *Bsal* (shown in red) were abundant throughout the genome, indicating diploidy. *Bsal* contigs were ordered according to normalized depth of read-coverage (Norm. DoC), and plotted against (from top to bottom) 1) Contig length in kb (shown as black dots). An enrichment of very small contigs were found over those with the lowest Norm. DoC, and we therefore excluded contigs < 5 kb. 2) the number of heterozygous calls were tallied according to 5 kb non-overlapping sliding windows (tallies shown as black dots) and the percent of them that were in tri-allelic probabilities (shown as a blue line). 3) In addition to bi-allelic (red), we also had a high number of positions that appeared tri-allelic (blue) or even tetra-allelic (green) across the genome. 4) The predominant allele frequency across the lowest covered 1/8th of the genome was tri-allelic, and the remainder of the genome was bi-allelic. **(b)** Synteny between *Bsal* and *Bd* reveals that only sub-chromosomal regions are predominantly tri-allelic (blue) compared with longer stretches that are red (di-allelic), suggesting *Bsal* has a diploid genome. **(c)** Repetitive element categories per megabase **(d)** Repetitive element categories stacked to show total portion of repetitive genome.

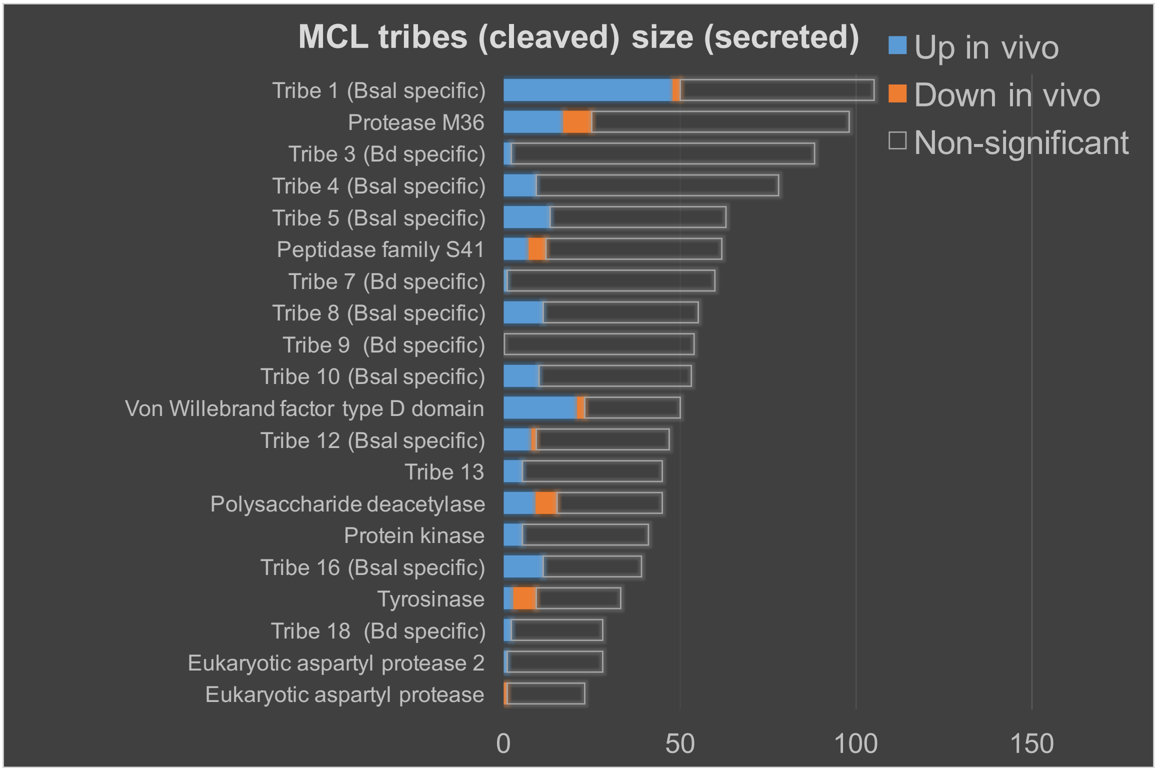

Supplementary Figure 3. Numbers of cleaved secreted genes for *Bd*, *Bsal*, *Hp* and *Sp* clustered into Markov Cluster Algorithm (MCL) tribes. Largest 20 tribes are shown, along with functional information or species-specificity where applicable. Colors signify significantly up- or down- regulated genes *in vivo*.

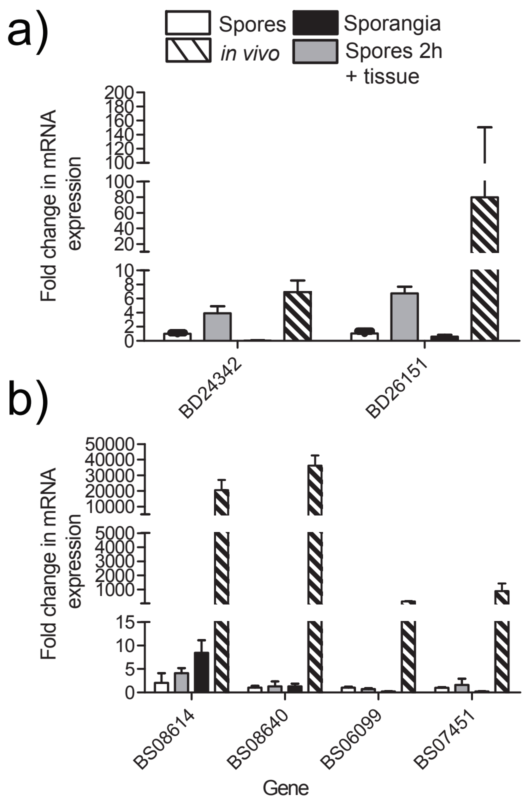

Supplementary Figure 4. Genes from Tribe 3 (a) and Tribe 1 (b) and their expression profile in *Bd* (above) and *Bsal* (below). Shown are the mean fold changes in mRNA expression of genes BD24342 and BD26151 in *Bd* and BS08614, BS08640, BS06099 and BS07451 in *Bsal*. The data represent the normalized target gene amount in spores that were incubated with skin tissue of *Tylototriton wenxianensis* (*Tw*) for 2 hours, 3 day old sporangia grown in TGhL, and skin tissue from chytrid-infected *Tw* animals relative to freshly collected spores which is considered 1. The results are presented as means + standard deviation. Significant differences in expression between each experimental group is shown in Table S6.

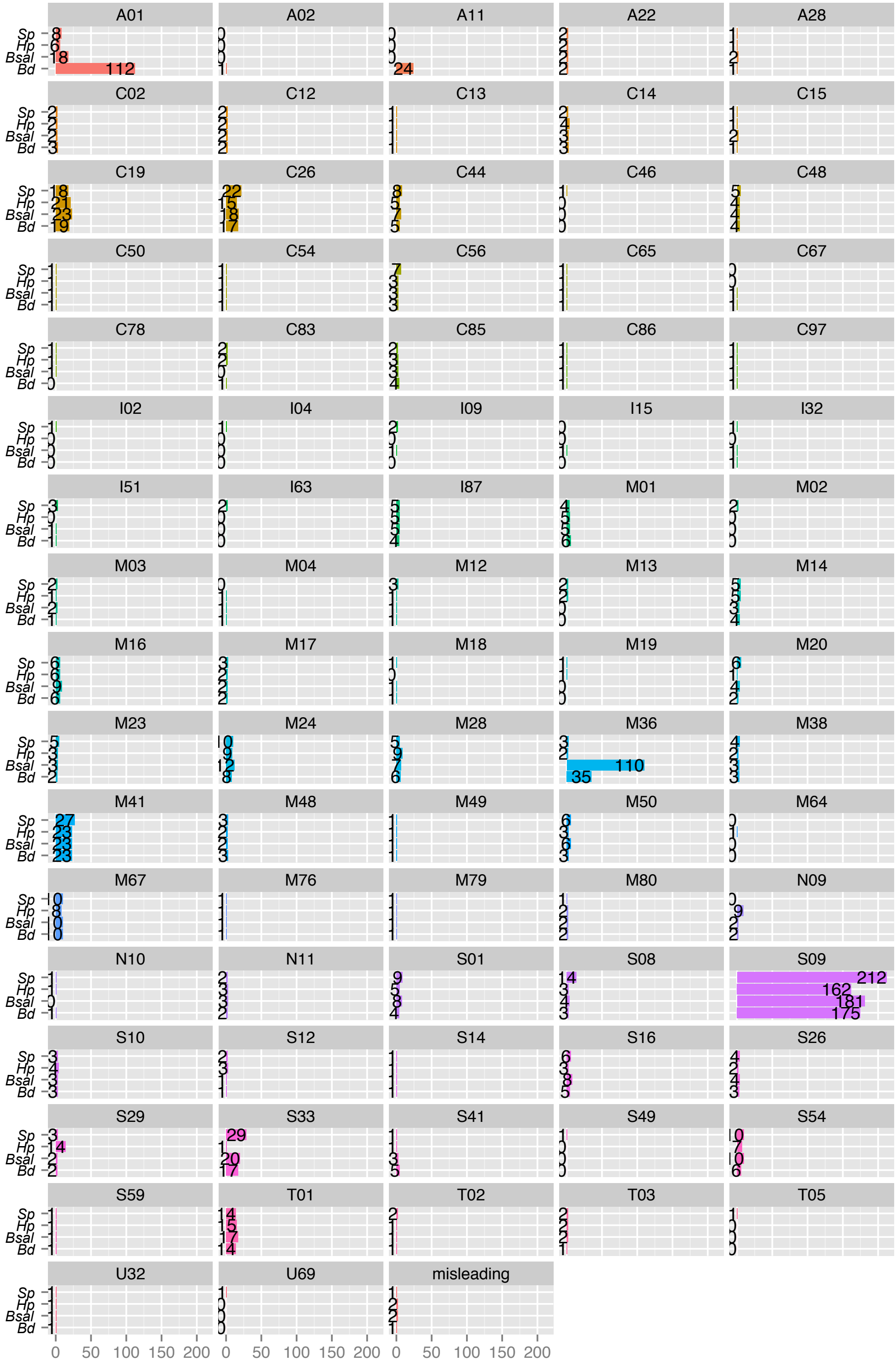

Supplementary Figure 5. The protease composition of each chytrid was determined using top high scoring pairs (HSPs) from BLASTp searches to the MEROPS database. Expansions of A01, and M36 were most noticeable in *Bd*, while expansions of M36 in *Bsal* were ~3X compared with the number in *Bd*.

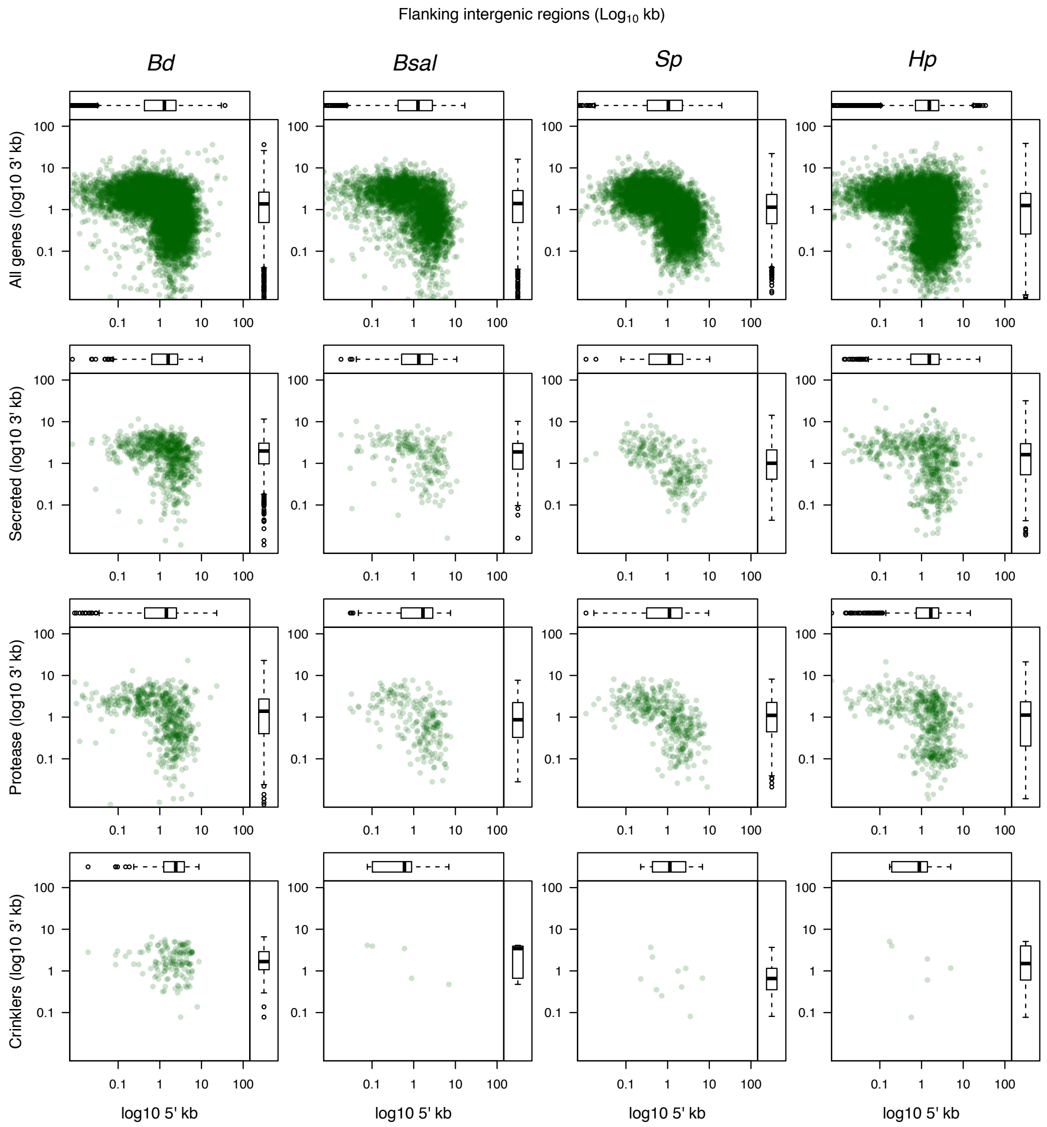

Supplementary Figure 6. Heat map showing the flanking intergenic regions (Log_{10} kb) for *Bd*, *Bsal*, *Sp* and *Hp* for four gene-subsets (all, secreted, proteases, crinklers). The x-axis shows the 5' upstream distance to the flanking gene, and the y-axis shows the 3' upstream distance to the flanking gene. Boxplots along the x-axis and y-axis for each gene set further summarize these values. Crinklers in *Bd* have longer intergenic regions compared with the other 3 categories.

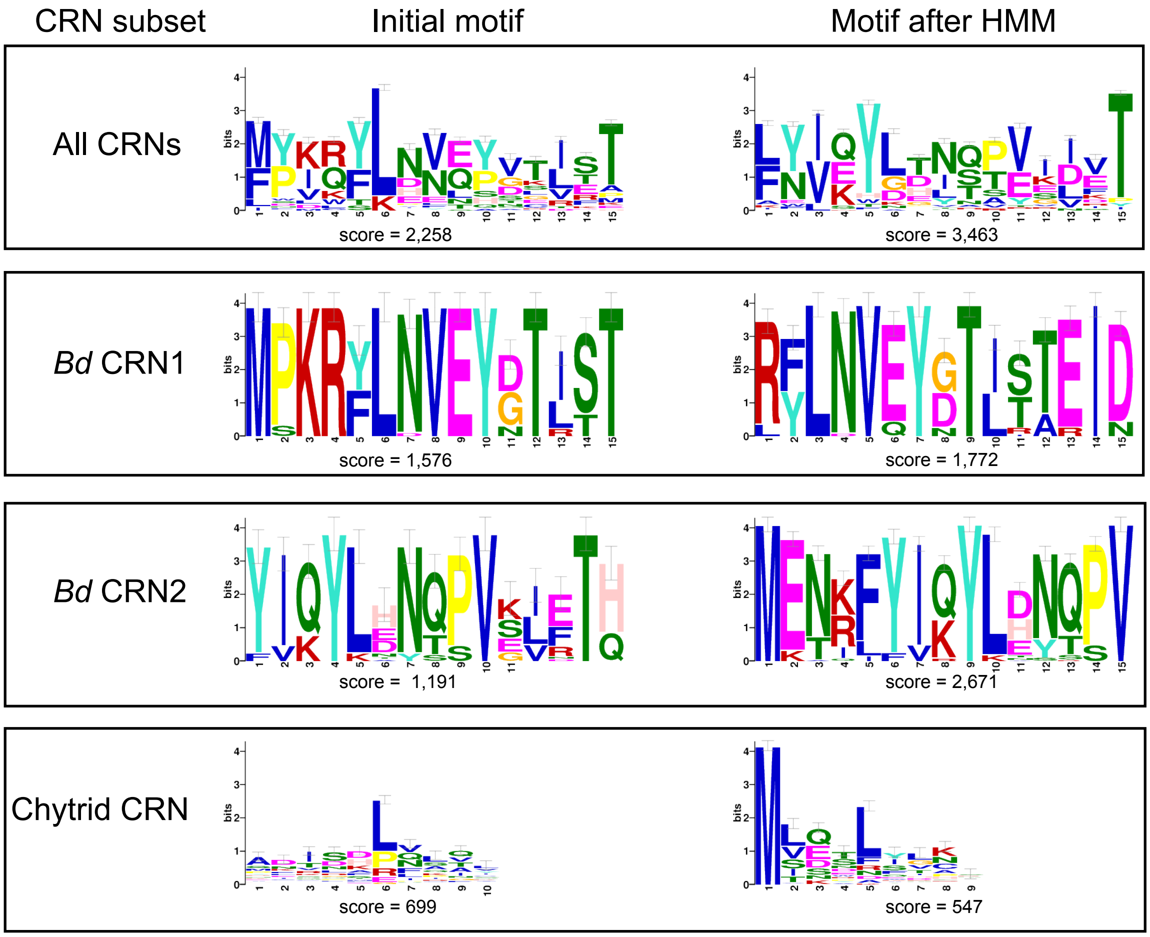

Supplementary Figure 7. Crinkler genes from *Bd* were screened for over-represented sequences using GLAM2. Crinklers frequently had 1 of 2 domains (CRN1 and CR2), which were manually split from the remaining CRNs that spanned all four chytrids (Chytrid CRN). These sequences were used to inform a hidden markov model (HMM), which we used to search for additional unidentified genes from the gene-sets, after which, new HMM were constructed. Each iteration achieved a slightly higher score.

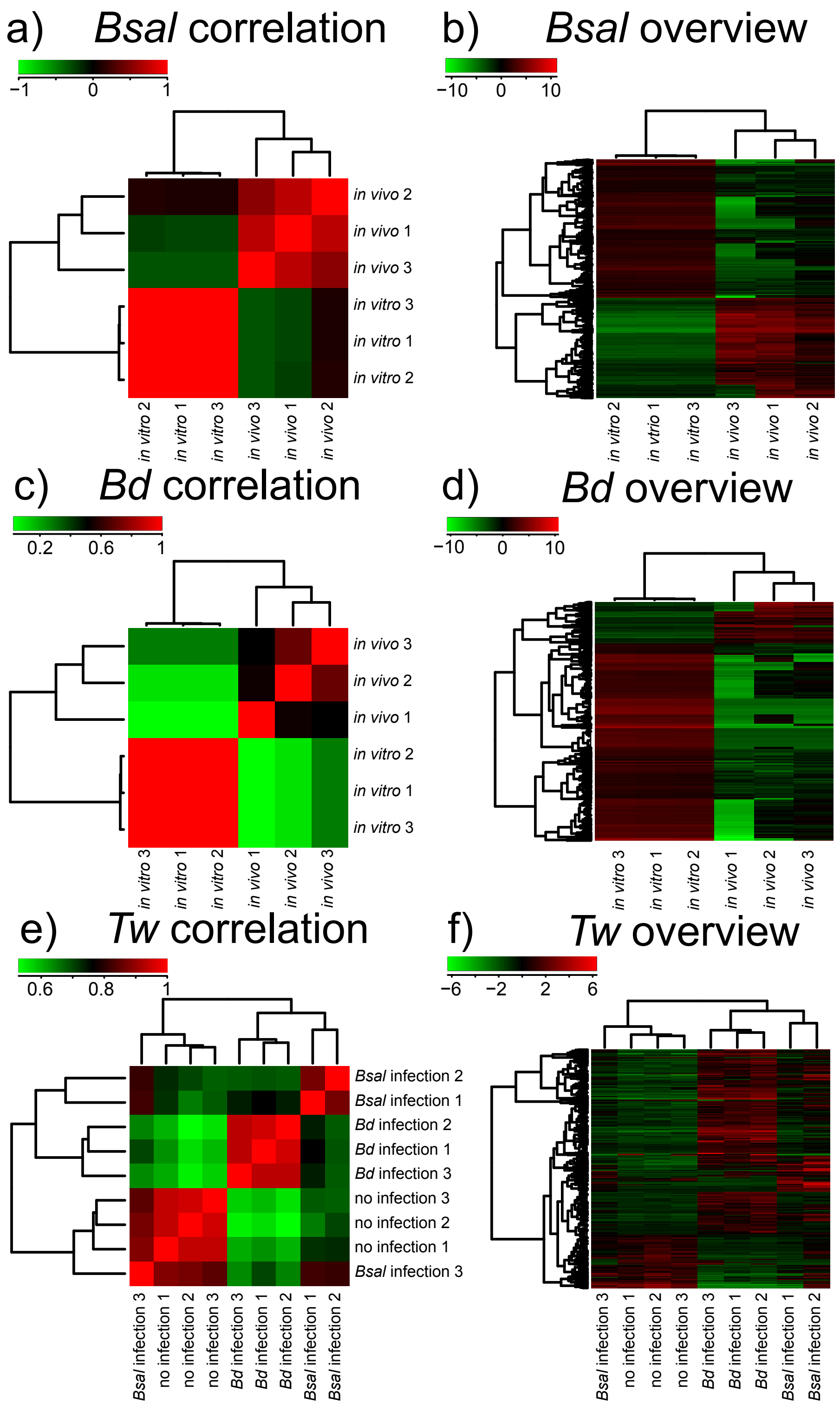

Supplementary Figure 8. The correlation of expression values between replicates and conditions of *Bsal* (a), *Bd* (c) and *Tw* (e) and an overview of all expression values for all differentially expressed genes in *Bsal* (b), *Bd* (d) and *Tw* (f). Trees indicate hierarchical clustering between datasets (above) and genes (left of heatmap).

Supplementary Tables

Supplementary Table 1. Details of genomic DNA and RNA from *Bd*, *Bsal* and *Tw* using 17 lanes of paired-end Illumina HiSeq and Illumina MiSeq. For the *Bsal* assembly, we sequenced to 182X depth, complementing with 868X deep transcriptome sequencing for guided gene-prediction. For the *Bden* assembly, a total of 12.5X Sanger sequence from 4 kb plasmid, 10 kb plasmid, and 40 kb Fosmid genomic clones is available in the Trace archive (center_project G888). For differential expression analysis of host and pathogens, we generated 780-1,224X depth of sequence.

Dataset adaptors or name	Dataset description	Sequencing description	Estimated depth (X)	Genbank accession
CGATGT_L005	*Bsal in vitro* DNA	29,503,468 paired-end HiSeq reads	182.6	PRJNA311566
23_S23_L001	*Bsal in vitro* DNA	5,895,159 paired-end MiSeq reads	80.3	PRJNA311566
GCCAAT_L007+CGATGT_L008+ATGTCA_L007	*Bsal in vitro* RNA (3 rep.)	78,103,411 paired-end HiSeq reads	868.9	PRJNA326249
ACAGTG_L007+TGACCA_L008+CTTGTA_L007	*Bd in vitro* RNA (3 rep.)	50,374,154 paired-end HiSeq reads	434.8	PRJNA326253
G89283+G89284+G89285	*Tw+Bd* infection RNA (3 rep.)	210,886,846 paired-end HiSeq reads	783.6*	PRJNA300849
G89280+G89281+G89282	*Tw+Bs* infection RNA (3 rep.)	235,040,547 paired-end HiSeq reads	829.1	PRJNA300849
G89277+G89278+G89279	*Tw in vitro* RNA (3 rep.)	240,725,021 paired-end HiSeq reads	1224	PRJNA300849

Supplementary Table 2. Genes and transcripts were predicted for *Bsal, Bd, Sp and Hp*. *Bsal* had a greater number of genes and transcripts, although a decrease in the proportion of spliced transcripts. *Bd* and *Bsal* had a similar mean number of exons per gene (*n*=~4.7), fewer than *Hp* (*n*=5) or *Sp* (*n*=6).

	Bsal	***Bd***	***Sp***	***Hp***
**genes #**	10,138	8,644	8,952	6,254
**transcripts #**	12,474	9,893	9,424	6,254
**spliced #**	9,875	8,940	8,322	5,347
**unspliced #**	2,599	953	1,102	907
**spliced transcripts (%)**	79.16	90.37	88.31	85.50
**exons #**	58,251	47,046	56,727	31,341
**introns #**	45,777	37,153	47,303	25,087
**Exons per gene #**	4.67	4.76	6.02	5.01

Supplementary Table 3. Genes from each of the four chytrids (*n*=33,974) were compared, and their orthogroups identified (*n*=13,056). The most abundant category (26%) was 1:1 orthologs between all four chytrids. A large number of genes were also unique to a species, or uniquely missing from one or more species.

**Type**	**Clusters**	**Genes in All 4 chytrids**	**Genes in *Sp***	**Genes in *Bsal***	**Genes in *Bd***	**Genes in *Hp***
missing in *Bd*	160	498	167	167	0	164
missing in *Bd* and *Bsal*	155	379	221	0	0	158
missing in *Bd* and *Bsal* and *Hp*	2884	3343	3343	0	0	0
missing in *Bd* and *Bsal* and *Sp*	983	1028	0	0	0	1028
missing in *Bd* and *Hp*	98	239	138	101	0	0
missing in *Bd* and *Hp* and *Sp*	1866	3300	0	3300	0	0
missing in *Bd* and *Sp*	75	242	0	167	0	75
missing in *Bsal*	95	330	102	0	127	101
missing in *Bsal* and *Hp*	34	77	41	0	36	0
missing in *Bsal* and *Hp* and *Sp*	935	1989	0	0	1989	0
missing in *Bsal* and *Sp*	65	211	0	0	134	77
missing in *Hp*	730	2428	812	821	795	0
missing in *Hp* and *Sp*	542	1356	0	671	685	0
missing in *Sp*	549	1820	0	612	592	616
1:1 orthologs	3378	13512	3378	3378	3378	3378
paralog in *Bd*	70	370	70	70	160	70
paralog in *Bd* and *Bsal*	37	347	37	79	194	37
paralog in *Bd* and *Bsal* and *Hp*	12	193	12	101	43	37
paralog in *Bd* and *Bsal* and *Sp*	19	139	40	40	40	19
paralog in *Bd* and *Hp*	9	54	9	9	18	18
paralog in *Bd* and *Hp* and *Sp*	4	29	8	4	9	8
paralog in *Bd* and *Sp*	10	69	28	10	21	10
paralog in *Bsal*	162	811	162	325	162	162
paralog in *Bsal* and *Hp*	4	25	4	9	4	8
paralog in *Bsal* and *Hp* and *Sp*	15	110	34	31	15	30
paralog in *Bsal* and *Sp*	16	97	33	32	16	16
paralog in *Hp*	36	182	36	36	36	74
paralog in *Hp* and *Sp*	7	43	15	7	7	14
paralog in *Sp*	83	431	182	83	83	83
paralog in all	23	322	80	85	86	71
**SUM**	13056	33974	8952	10138	8630	6254

Supplementary Table 4. Two-tailed Fisher's exact test with *q*-value FDR for PFAM domains enriched in the orthogroups unique to *Bd and Bsal*

Pfam domain	*Bsal+Bden* count	Other count	fisher p-value	q-value FDR
PF00665.21 Integrase core domain	19	10	2.23E-19	6.86E-16
PF01522.16 Polysaccharide deacetylase	23	27	2.02E-18	3.12E-15
PF05699.9 hAT family C-terminal dimerisation region	9	4	4.10E-10	4.21E-07
PF00187.14 Chitin recognition protein	10	8	9.38E-10	7.22E-07
PF00264.15 Common central domain of tyrosinase	11	17	1.40E-08	8.60E-06
PF12767.2 Transcriptional regulator of RNA polII, SAGA, subunit	5	0	1.76E-07	9.06E-05
PF00096.21 Zinc finger, C2H2 type	14	45	2.25E-07	9.92E-05
PF04674.7 Phosphate-induced protein 1 conserved region	7	7	9.02E-07	3.47E-04
PF03732.12 Retrotransposon gag protein	4	0	3.97E-06	1.22E-03
PF12776.2 Myb/SANT-like DNA-binding domain	4	0	3.97E-06	1.22E-03
PF01393.14 Chromo shadow domain	5	3	8.82E-06	2.27E-03
PF14543.1 Xylanase inhibitor N-terminal	5	3	8.82E-06	2.27E-03
PF00188.21 Cysteine-rich secretory protein family	7	15	3.28E-05	7.76E-03
PF00335.15 Tetraspanin family	5	5	3.68E-05	8.10E-03
PF02265.1 S1/P1 Nuclease	6	10	4.26E-05	8.75E-03
PF00615.14 Regulator of G protein signaling domain	8	23	4.83E-05	9.31E-03
PF00503.15 G-protein alpha subunit	7	17	6.15E-05	1.11E-02
PF00444.13 Ribosomal protein L36	3	0	8.92E-05	1.19E-02
PF01315.17 Aldehyde oxidase and xanthine dehydrogenase, a/b hammerhead domain	3	0	8.92E-05	1.19E-02
PF02892.10 BED zinc finger	3	0	8.92E-05	1.19E-02
PF03450.12 CO dehydrogenase flavoprotein C-terminal domain	3	0	8.92E-05	1.19E-02
PF06390.7 Neuroendocrine-specific golgi protein P55 (NESP55)	3	0	8.92E-05	1.19E-02
PF14372.1 Domain of unknown function (DUF4413)	3	0	8.92E-05	1.19E-02
PF13465.1 Zinc-finger double domain	9	34	9.71E-05	1.25E-02
PF00067.17 Cytochrome P450	6	16	3.16E-04	3.89E-02

Supplementary Table 5. List of the M36 genes and their categories in each chytrid.

Bsal G1M36	*Bsal* G2M36	*Bd* G1M36	Chytrid Conserved
BS_00574	BS_01185	BDEG_20379	BDEG_22774
BS_01765	BS_04588	BDEG_20405	BDEG_22775
BS_02529	BS_05326	BDEG_21659	BDEG_23332
BS_03474	BS_05417	BDEG_22285	BDEG_24855
BS_04080	BS_05418	BDEG_23049	BDEG_25147
BS_04296	BS_06029	BDEG_23209	BDEG_28447
BS_04381	BS_06539	BDEG_23812	BS_00669
BS_04580	BS_06629	BDEG_23816	BS_02495
BS_05919	BS_06630	BDEG_23888	BS_05830
BS_05933	BS_06778	BDEG_24460	BS_07719
BS_05934	BS_07154	BDEG_24587	Hp_00506
BS_06103	BS_07519	BDEG_24892	Hp_0229
BS_06223	BS_07596	BDEG_25168	SPPG_01665
BS_06699	BS_07600	BDEG_25206	SPPG_02641
BS_06708	BS_07631	BDEG_25536	SPPG_04344
BS_06905	BS_07642	BDEG_25875	
BS_07250	BS_07775	BDEG_25883	
BS_07695	BS_07805	BDEG_25898	
BS_08058	BS_07997	BDEG_27285	
BS_08136	BS_08134	BDEG_27471	
BS_08438	BS_08194	BDEG_27850	
BS_08686	BS_08285	BDEG_27916	
BS_08742	BS_08289	BDEG_27920	
BS_08935	BS_08364	BDEG_27923	
BS_08963	BS_08381	BDEG_27937	
BS_09156	BS_08396	BDEG_28290	
BS_09225	BS_08444	BDEG_28609	
BS_09282	BS_08522		
BS_09289	BS_08534		
BS_09338	BS_08610		
BS_09371	BS_08717		
BS_09541	BS_08732		
BS_09557	BS_08753		
BS_09566	BS_08767		
BS_09677	BS_08786		
BS_09687	BS_08816		
BS_09704	BS_09094		
BS_09721	BS_09097		
BS_09779	BS_09112		
BS_09821	BS_09203		
BS_09876	BS_09213		
BS_09892	BS_09520		
BS_10047	BS_09537		
BS_10090	BS_09558		
BS_10154	BS_09568		
	BS_09612		
	BS_09613		
	BS_09672		
	BS_09749		
	BS_09805		
	BS_09935		
	BS_09972		
	BS_09985		
	BS_10015		
	BS_10040		
	BS_10079		
	BS_10093		

Supplementary Table 6: **Gene expression for different life stages.** The numerical values shown indicate fold change ± standard deviation in comparison with freshly collected spores. Superscript refers to a significant difference with the respective condition being “A” spores, “B” spores 2h + tissue, “C” sporangia, “D” sporangia + chitinase, and “E” *in vivo*. Gene targets included Group 1 BatraM36s (G1M36) and Group 2 BsalM36s (G2M36), Carbohydrate binding-module 18 (CBM18), Crinklers (CRN), and Tribes with unknown function (extra). A Kruskal-Wallis analysis, followed by a Dunn-Bonferroni post hoc test was performed, indicating significant changes with $p < 0.05$. NA indicates that the condition was not tested.

		A: Spores	B: Spores 2h + tissue	C: Sporangia	D: Sporangia + chitinase	E: *In vivo*
Bd G1M36	G1M_20379	1.01 ± 0.19 ^{C}	0.37 ± 0.10 ^{E}	0.16 ± 0.08 ^{A,E}	NA	27.36 ± 11.88 ^{B,C}
	G1M_23888	1.03 ± 0.27 ^{C}	0.31 ± 0.16	0.02 ± 0.01 ^{A,E}	NA	1.24 ± 0.50 ^{C}
	G1M_27285	1.04 ± 0.32 ^{B}	0.01 ± 0.00 ^{A,E}	0.02 ± 0.01 ^{E}	NA	3.43 ± 2.61 ^{B,C}
	G1M_25206	1.28 ± 1.06	2.94 ± 1.27 ^{C}	0.26 ± 0.14 ^{B,E}	NA	12.52 ± 11.93 ^{C}
	G1M_22285	1.01 ± 0.17	0.63 ± 0.19 ^{E}	0.60 ± 0.15 ^{E}	NA	3.66 ± 1.91 ^{B,C}
Bsal G1M/G2M	G2M_08289	1.03 ± 0.27 ^{C}	0.25 ± 0.07 ^{E}	0.01 ± 0.00 ^{A,E}	NA	5.17 ± 3.29 ^{B,C}
	G2M_08767	1.23 ± 0.74 ^{E}	3.96 ± 2.89	1.65 ± 0.64 ^{E}	NA	200.53 ± 69.69 ^{A,C}
	G2M_08444	1.04 ± 0.32	0.15 ± 0.05 ^{E}	0.09 ± 0.05 ^{E}	NA	111.16 ± 46.85 ^{B,C}
	G1M_06223	1.06 ± 0.41 ^{C}	12.17 ± 1.59 ^{E}	29.71 ± 24.37 ^{A,E}	NA	0.95 ± 0.32 ^{B,C}
	G1M_04381	1.34 ± 1.18 ^{E}	2.16 ± 0.85	1.28 ± 0.38 ^{E}	NA	2099.07 ± 914.56 ^{A,C}
Bd CBM18	CBM18_20255	1.01 ± 0.13 ^{C,D}	1.69 ± 0.43 ^{C,D}	0.02 ± 0.00 ^{A,B}	0.02 ± 0.01 ^{A,B}	0.48 ± 0.15
	CBM18_28751	1.17 ± 0.69 ^{B}	0.09 ± 0.14 ^{A,E}	0.18 ± 0.09 ^{E}	0.24 ± 0.14 ^{E}	15.77 ± 7.88 ^{B,C,D}
	CBM18_26087	1.43 ± 1.53 ^{B,E}	14.06 ± 4.91 ^{A,C,D}	1.72 ± 0.36 ^{B}	2.11 ± 0.79 ^{B}	7.13 ± 1.12 ^{A}
Bsal CBM18	CBM18_04331	1.08 ± 0.43	1.87 ± 0.56 ^{D}	0.19 ± 0.06 ^{E}	0.18 ± 0.07 ^{B,E}	37.56 ± 9.07 ^{C,D}
	CBM18_07447	1.01 ± 0.11 ^{E}	4.03 ± 1.13 ^{D}	1.20 ± 0.35	0.41 ± 0.11 ^{B,E}	159.13 ± 84.22 ^{A,D}
	CBM18_08642	1.01 ± 0.15 ^{C,D}	1.70 ± 0.32	4.04 ± 1.24 ^{A,E}	4.20 ± 0.78 ^{A,E}	0.15 ± 0.16 ^{C,D}
Bd CRN	CRN_23176	1.01 ± 0.16 ^{C}	20.98 ± 1.72 ^{C,E}	0.05 ± 0.02 ^{A,B}	NA	0.10 ± 0.03 ^{B}
	CRN_25085	1.02 ± 0.21 ^{C}	8.14 ± 1.08 ^{C,E}	0.03 ± 0.02 ^{A,B}	NA	0.17 ± 0.15 ^{B}
	CRN_22492	1.04 ± 0.28 ^{C}	6.73 ± 0.94 ^{C,E}	0.02 ± 0.02 ^{A,B}	NA	0.12 ± 0.07 ^{B}
Bsal CRN	CRN_00955	1.03 ± 0.27 ^{C,E}	0.19 ± 0.09 ^{C}	0.00 ± 0.00 ^{A,B}	NA	0.02 ± 0.00 ^{A}
	CRN_06851	1.01 ± 0.14 ^{C,E}	0.22 ± 0.08 ^{C}	0.00 ± 0.00 ^{A,B}	NA	0.06 ± 0.03 ^{A}
Bd extra	BD_24342	1.01 ± 0.13 ^{E}	3.93 ± 0.98 ^{C}	0.04 ± 0.04 ^{B,E}	NA	6.91 ± 1.65 ^{A,C}
	BD_26151	1.05 ± 0.34 ^{E}	6.75 ± 0.94 ^{C}	0.61 ± 0.22 ^{B,E}	NA	79.67 ± 70.73 ^{A,C}
Bsal extra	BS_08614	2.07 ± 2.04 ^{E}	4.15 ± 1.06 ^{E}	8.47 ± 2.65	NA	20578.81 ± 6426.80 ^{A,B}
	BS_08640	1.04 ± 0.34 ^{E}	1.30 ± 1.04 ^{E}	1.31 ± 0.56	NA	36140.74 ± 6524.31 ^{A,B}
	BS_06099	1.02 ± 0.20 ^{C}	0.74 ± 0.19 ^{E}	0.18 ± 0.08 ^{A,E}	NA	148.43 ± 39.01 ^{B,C}
	BS_07451	1.01 ± 0.13	1.62 ± 1.33	0.19 ± 0.06 ^{E}	NA	888.55 ± 547.44 ^{C}

Supplementary Table 7. Two-tailed *t-tests* were calculated on 5' and 3' intergenic distances of genes split between the four chytrids, and further separated secreted, proteases, and crinklers. Green highlighting signifies $p<0.0001$.

5' log10

	Full set (Bd)	Full set (bsal)	Full set (Sp)	Full set (Hp)	secreted (Bd)	protease (Bd)	crinklers (Bd)	secreted (Bsal)	protease (Bsal)	crinklers (Bsal)	secreted (Sp)	protease (Sp)	crinklers (Sp)	secreted (Hp)	protease (Hp)
**Full set (Bd)**															
**Full set (bsal)**	0.535														
**Full set (Sp)**	3.67E-08	0.0003597													
**Full set (Hp)**	1.18E-15	1.94E-10	< 2.2e-16												
**secreted (bd)**	4.76E-07	6.01E-07	7.15E-14	0.1204											
**protease (bd)**	0.6664	0.491	0.01317	0.02185	0.004119										
**crinklers (bd)**	2.57E-14	1.71E-14	< 2.2e-16	1.16E-09	8.85E-07	7.49E-11									
**secreted (Bsal)**	0.8257	0.7105	0.1855	0.2183	0.07953	0.9932	1.20E-06								
**protease (Bsal)**	0.1394	0.1029	0.005187	0.7729	0.3295	0.305	4.44E-06	0.4367							
**crinklers (Bsal)**	0.4651	0.476	0.5466	0.3733	0.3382	0.45	0.1732	0.4522	0.3884						
**secreted (Sp)**	0.4664	0.62	0.4163	0.006259	0.001281	0.3898	8.20E-11	0.5414	0.1061	0.5033					
**protease (Sp)**	0.04966	0.09892	0.853	2.16E-05	5.57E-06	0.06869	4.60E-14	0.2139	0.01635	0.5565	0.4502				
**crinklers (Sp)**	0.6236	0.5919	0.4169	0.9536	0.8937	0.6753	0.188	0.6818	0.8998	0.385	0.5276	0.4042			
**secreted (Hp)**	0.06527	0.04254	0.000132	0.4057	0.09649	0.2957	1.19E-08	0.4865	0.8245	0.4006	0.08398	0.005823	0.8482		
**protease (Hp)**	0.007889	0.005076	4.57E-06	0.977	0.3462	0.09622	2.01E-07	0.2682	0.7947	0.3729	0.02497	0.0009391	0.9579	0.5343	
**crinklers (Hp)**	0.6727	0.6948	0.835	0.4884	0.4201	0.6425	0.1407	0.6474	0.5197	0.6788	0.7492	0.8536	0.528	0.5432	0.5432

3' log10

	Full set (Bd)	Full set (bsal)	Full set (Sp)	Full set (Hp)	secreted (Bd)	protease (Bd)	crinklers (Bd)	secreted (Bsal)	protease (Bsal)	crinklers (Bsal)	secreted (Sp)	protease (Sp)	crinklers (Sp)	secreted (Hp)	protease (Hp)
**Full set (Bd)**															
**Full set (bsal)**	0.5586														
**Full set (Sp)**	2.65E-07	0.0012													
**Full set (Hp)**	< 2.2e-16	< 2.2e-16	1.14E-12												
**secreted (bd)**	< 2.2e-16	6.55E-16	< 2.2e-16	< 2.2e-16											
**protease (bd)**	0.3204	0.4941	0.3891	0.0003388	2.08E-09										
**crinklers (bd)**	3.20E-10	2.63E-10	5.93E-14	< 2.2e-16	0.1775	7.68E-09									
**secreted (Bsal)**	0.003706	0.002606	4.56E-05	8.00E-09	0.2774	0.002495	0.05745								
**protease (Bsal)**	0.000588	0.001238	0.01732	0.4237	1.81E-10	0.01105	5.83E-11	5.50E-06							
**crinklers (Bsal)**	0.3432	0.3307	0.2651	0.1802	0.7466	0.3003	0.902	0.6115	0.1494						
**secreted (Sp)**	0.06123	0.1102	0.7131	0.06486	2.34E-09	0.393	2.34E-09	0.0004965	0.07891	0.25					
**protease (Sp)**	0.01155	0.02817	0.3814	0.1208	7.66E-12	0.2044	5.14E-11	9.84E-05	0.1219	0.2316	0.7428				
**crinklers (Sp)**	0.1732	0.1855	0.2692	0.4836	0.03557	0.2234	0.02219	0.05894	0.6353	0.1183	0.3048	0.3414			
**secreted (Hp)**	0.1939	0.1362	0.002025	1.07E-08	6.01E-05	0.09525	1.78E-05	0.08585	0.0002349	0.4123	0.02029	0.004884	0.127		
**protease (Hp)**	1.09E-07	1.08E-06	0.0002576	0.2203	< 2.2e-16	0.0006263	< 2.2e-16	2.75E-08	0.9982	0.149	0.02432	0.04296	0.6285	7.72E-07	
**crinklers (Hp)**	0.9266	0.9082	0.8028	0.6316	0.6705	0.8604	0.5673	0.7816	0.5571	0.583	0.7741	0.7398	0.4465	0.9839	0.9839

Supplementary Table 8. Trinity assemblies for the RNA-Seq data from *Tw, Bsal and Bd* combined datasets were made using Kmer depth=2 setting. Kmer depth=1 setting was also tested, but achieved considerably shorter assembled contigs in terms of NMAX, N50 and N90. We ran TransDecoder to identify predicted reading frames from the assembly, and then used BLAT to identify *Batrachochytrium* transcripts from their respective genomes, splitting the dataset into 4 categories (*Batrachochytrium*-non-specific, *Bd* only, *Bsal* only, *Tw* only).

Assembly and alignment metrics	kmer depth=1	kmer depth=2
Trinity number of sequences	732,326	437,048
Trinity length (nt)	486,010,742	353,024,040
Trinity NMAX:	28,751	65,583
Trinity N50:	1,162	1,791
Trinity N90:	252	275
TransDecoder number of sequences	123,247	94104
TransDecoder length (nt)	103,499,169	88802019
TransDecoder NMAX:	28,749	65310
TransDecoder N50:	1,251	1440
TransDecoder N90:	351	369
BLAT to *Bd* genome (nt covered from all hits)	5,594,034	2248563
BLAT to *Bd* genome (nt covered from all hits over ORFs)	3,281,696	1402322
BLAT to *Bd* Genes covered (by >=50)	2456 (27.85%)	1103 (12.51%)
BLAT to *Bd* Genes not covered	6,363	7716
BLAT to *Bd* Coverage over non-predicted ORF (number)	9,998	4145
BLAT to *Bd* Coverage over non-predicted ORF (nt)	2,312,337	846241
BLAT to *Bsal* genome (nt covered from all hits)	10,885,989	7855982
BLAT to *Bsal* genome (nt covered from all hits over ORFs)	7,272,260	5144596
BLAT to *Bsal* Genes covered (by >=50)	4616 (37.00%)	3381 (27.10%)
BLAT to *Bsal* Genes not covered	7,858	9093
BLAT to *Bsal* Coverage over non-predicted ORF (number)	22,147	16218
BLAT to *Bsal* Coverage over non-predicted ORF (nt)	3,613,266	2711049
Bd + *Bsal* TransDecoder transcripts number	197	155
Bd + *Bsal* TransDecoder transcripts length (nt)	171465	140892
GC (%)	47	48
NMAX	3894	3591
N50	1092	1218
N90	432	411
Bd only TransDecoder transcripts number	3163	1390
Bd only TransDecoder transcripts length (nt)	1817508	796167
GC (%)	45	46
NMAX	5406	4788
N50	597	585
N90	336	333
Bsal only TransDecoder transcripts number	10013	7438
Bsal only TransDecoder transcripts length (nt)	8274402	5835474
GC (%)	47	48
NMAX	10929	10929
N50	1047	969
N90	402	387
Tw (not chytrid) TransDecoder transcripts number	110,268	85121
Tw (not chytrid) TransDecoder transcripts length (nt)	93578724	82029486
GC (%)	48	48
NMAX	28749	65310
N50	1305	1509
N90	348	369

Supplementary Table 9. *Tw* gene counts that were significantly up- or down- regulated without infection (control), infected with *Bsal*, and infected with *Bd*. Gene counts are followed by the number of PFAM and GO terms associated with those genes, and their respective enrichment as determined by Two-tailed Fisher's exact tests with *q*-value FDR.

Experiment	UP/Down-regulated	Genes #	Genes with PFAM domains	Genes with GO terms	PFAM enrichment	GO enrichment
Tw infected with Bsal	upregulated	96	46	31	27	0
Tw infected with Bsal	downregulated	12	5	3	4	0
Tw infected with Bd	upregulated	384	175	103	73	2
Tw infected with Bd	downregulated	106	48	46	52	0

Supplementary Table 10: List of genes and sequences of the primers used for quantitative PCR analysis.

Gene	primer	primer sequence (5' - 3')
Bd: α-centractin	sense	GCAGCATGGAGTTGTCACTG
	antisense	AGCTTGGTCACGATTGGAAC
Bd: R6064	sense	GTCGTACTGGCAACCTCACC
	antisense	ACATTGGGAGCAATCTCGAC
Bd: TEF1a	sense	CCTTCCCGTCCTACTGACAA
	antisense	GAACAGTTCCGATTCCTCCA
Bd: GAPDH	sense	AAGCCTGCCAAGTACGAAGA
	antisense	AAAGATGGAGCTGCGAGTGT
Bd: G1M_20379	sense	CTGGTATGGACGCTCTCGTT
	antisense	AGACTAAGCCAGTCGCTCCA
Bd: G1M_23888	sense	CACCCAACGAGTTCAAGGTT
	antisense	GCCGTCTTGGATATGGACAG
Bd: G1M_27285	sense	GCAGATGGTCAACCTGGAGT
	antisense	CCACTGTCTCGATTCGGATT
Bd: G1M_25206	sense	GGTCTTGACAGCCAAATCGT
	antisense	CTCTAGCCTCACCGTCGAAC
Bd: G1M_22285	sense	GGTGTCTCAGGTCGGTTGAC
	antisense	GATTCCTTGGCAGACACGAT
Bd: CBM18_20255	sense	ATCTTGCTTGACACCCGAAG
	antisense	GTGACTTGGCTGATGCCTTT
Bd: CBM18_28751	sense	CGTGTGGACGTCGATACAAC
	antisense	CATCCAACACTGCAATGAGC
Bd: CBM18_26087	sense	TCGTGAACTAACGCAACAGC
	antisense	CAGACGGTACTTGACGCAGA
Bd: BDEG_24342	sense	CCGGCTACAAGCTTGTGAGA
	antisense	GTGTTGGATCCAGGACCCTG
Bd: BDEG_26151	sense	CAGCTGATGAAGATGGCTCA
	antisense	GGTTCGTTAGTCGGGACAGA
Bd: CRN_23176	sense	AAACGCCCTTCGCTTCGATA

	antisense	TCTTTCTCCAAGCTGAGCGG
Bd: CRN_25085	sense	CTCCCGGTTCGACATCACAA
	antisense	GAACAGCGAACCACAGCTTG
Bd: CRN_22492	sense	CTCCCGGTTCGACATCACAA
	antisense	GAACAGCGAACCACAGCTTG
Bsal: α-centractin	sense	CCGGCTACCATTTTCATACG
	antisense	CGATCGATGGGTAGCACTCT
Bsal: R6064	sense	GTTGCCAAGTCTGCTGTGAA
	antisense	ATCAAGCGAGGGTGCAGAC
Bsal: TEF1a	sense	TCCCACTGACAAACCTCTCC
	antisense	CGACAGGTACTGTTCCAATACCAC
Bsal: GAPDH	sense	GCCAGCAAAATACGAGGAGA
	antisense	CCATTCATGGGTCCATTAGC
Bsal: G2M_08289	sense	GTCATCCTGGCGTGCTAAAT
	antisense	CACCAGTAAGGCGGTCAGAT
Bsal: G2M_08767	sense	ACGAGCGACGAGCATATACC
	antisense	CACCAGTGTTTGCATTGACC
Bsal: G2M_08444	sense	CTGTGATGGGGAGCTATTGG
	antisense	GAAACGATACCGCCAGTGTT
Bsal: G1M_06223	sense	CAGTTCCACGTCGTGATGTC
	antisense	TCCTTTGATCTCAGGGTTGG
Bsal: G1M_04381	sense	TGATGGACAACCAGGAGTGA
	antisense	CCATGTGCATACTCGTGGAG
Bsal: CBM18_04331	sense	TCCATATGATCTCGGGTGGT
	antisense	CAGCAGCGTCAATAGTCCAA
Bsal: CBM18_07447	sense	ATACCGATACGCCTGTTGGA
	antisense	ACAGAAGCCAGTTGGTGAGC
Bsal: CBM18_08642	sense	CAGCACCCAACCCTAAGATG
	antisense	CAAAGAGACCGGGACTGGTA
Bsal: BS_08614	sense	CGTTGGAGATGGTGTTGTTG
	antisense	CAGGGCCATCAATCTTCTGT
Bsal: BS_08640	sense	GACAAGGCCGAACTTGGTT
	antisense	CGGGCATTTCAACATACTCC
Bsal: BS_06099	sense	TATGTGCTGCTGCCATTGGA
	antisense	CTTGCTGTTGAGCTTGGCTG
Bsal: BS_07451	sense	TTTGCACTGCAGACCTCAAG
	antisense	GGCACCTGGTGTTTTGTTCT
Bsal: CRN_00955	sense	TATGATGGGAAGCCTCAGGA
	antisense	TCGCCCTTTACAATCTCGTC
Bsal: CRN_06851	sense	GAAATGGGTGGAATGGACAC
	antisense	AAGTTGGCCCTCTTGGAATC
